# General practice and ethnicity: an experimental study of doctoring

**DOI:** 10.1186/1471-2296-15-89

**Published:** 2014-05-09

**Authors:** Brice Lepièce, Christine Reynaert, Philippe van Meerbeeck, Vincent Lorant

**Affiliations:** 1 Institute of Health and Society, Université Catholique de Louvain, Clos Chapelle-aux-Champs 30, box 3016–1200, Brussels, Belgium

**Keywords:** Health inequality, General practice, Medical decisions, Doctor-patient relationship, Ethnic discrimination

## Abstract

**Background:**

There is extensive evidence of health inequality across ethnic groups. Inequity is a complex social phenomenon involving several underlying factors, including ethnic discrimination. In the field of health care, it has been established that ethnic discrimination stems partially from bias or prejudice on the part of doctors. Indeed, it has been hypothesized that patient ethnicity may affect doctors’ social cognition, thus modifying their social interactions and decision-making processes. General practitioners (GPs) are the primary access point to health care for ethnic minority groups. In this study, we examine whether patient ethnicity affects the relational and decisional features of doctoring.

**Methods:**

The sample was made up of 171 Belgian GPs, who were each randomly allocated to one of two experimental conditions. One group were given a hypertension vignette case with a Belgian patient (non-minority patient), while the other group were given a hypertension vignette case with a Moroccan patient (minority patient). We evaluated the time devoted by GPs to examining medical history; time devoted by GPs to examining socio-relational history; cardiovascular risk assessments by GPs; electrocardiogram (ECG) recommendations by GPs, and drug prescriptions by GPs.

**Results:**

We observed that for ethnic minority patients, GPs prescribed more drugs and devoted less time to examining socio-relational history. Neither cardiovascular risk assessments nor ECG recommendations were affected by patient ethnicity. GPs who were very busy devoted less time to examining medical history when dealing with minority patients.

**Conclusions:**

We found no evidence that GPs discriminated against ethnic minority patients when it came to medical decisions. However, our study did identify a risk of drugs being used inappropriately in some ethnic-specific encounters. We also observed that, with ethnic minority patients, GPs engage less in the relational dimension of doctoring, particularly when working within a demanding environment. In general practice, the quality of the relationship between doctor and patient is an essential component of the effective management of chronic illness. Our research highlights the complexity of ethnic discrimination in general practice, and the need for further studies.

## Background

Inequalities in health status for ethnic minority groups are well documented, with ethnic minorities consistently faring worse in terms of morbidity and mortality [1-3]. Moreover, in recent years, the gap in mortality rates has widened critically for many illnesses [4].

Many factors have been suggested to explain these health inequalities. Shavers listed the factors most commonly assessed in research: access to health care; socio-economic status; lack of knowledge of the health care system; cultural beliefs or preferences, and race/ethnicity-based discrimination [5].

Smedley concluded that ethnic discrimination existed at all levels of health care, from the policy level to the level of the individual provider [6]. Smedley’s research showed that for a wide range of medical procedures, ranging from highly technological interventions to basic diagnostic and treatment procedures, minority patients are less likely to receive adequate medical procedures and more likely to experience a poorer quality of medical care than non-minority patients. This pattern of differences remained true even when studies adjusted for differences in patients’ health insurance, socio-economic status, the stage and severity of the disease, and comorbidity.

Pager suggested three major mechanisms as underlying contemporary forms of ethnic discrimination: the individual level, the organizational level, and the structural level [7]. In this paper, we focus on the individual level of ethnic discrimination in health care. According to van Ryn, negative stereotypes that doctors have about ethnic minorities are likely to play a role in discrimination during medical encounters [8]. Explicit and implicit biases on behalf of providers can lead to discrimination in health care for ethnic minorities and, ultimately, to disparities in health [9-12]. These stereotypes negatively influence the course and the outcomes of clinical encounters and lead to distrust within inter-ethnic medical interactions [11,13].

Tajfel described “in-group bias”*,* and showed, through a set of pioneering experiments, that humans tend to favor in-group members over out-group members in the distribution of rewards, even when these groups are arbitrarily categorized [14]. The idea is that we like, and are more motivated to help, people that we think are like us [9]. Even though doctors are bound by professional and ethical standards to help and care for vulnerable patients, they may unknowingly stereotype members of ethnic minorities, particularly when working under conditions that diminish cognitive capacity, such as time pressure, fatigue, and information overload [15].

There is extensive evidence to indicate that patient ethnicity modifies conscious and unconscious beliefs and practices on the part of doctors [16-19]. Doctors may be unaware of the fact that they hold implicit attitudes and stereotypes about ethnicity, but these implicit social biases can significantly influence quality of care [19]. According to van Ryn, these biases affect doctors when it comes to decision-making, interactions, and inter-personal behaviors [8]. For example, there is evidence that doctors contribute to ethnicity inequities in access to kidney transplants, cardiac procedures, psychiatric care, and pain control [8]. Ethnic minority patients are also more likely than non-minority patients to be recommended for unnecessary surgery [20]. Another major dimension of doctoring that is affected in inter-ethnic consultations is communication and the doctor-patient relationship [21]. For example, Roter revealed that when dealing with black patients, American physicians were more likely to adopt a “narrowly biomedical” communication pattern, characterized by low levels of patient control over communication, low levels of psychosocial talk, and high levels of biomedical information-giving [22]. Johnson found that doctors were less patient-centered, more verbally dominant, and expressed less positive affect with African-American patients compared to white patients [23]. Kaplan and Cooper found that minority patients rated their physician as having a less participatory decision-making style than did non-minority patients [24,25].

There has been little research focusing on ethnic discrimination in general practice. Indeed, most studies on this topic have been conducted within the field of Specialized Medicine. However, GPs are often the primary access to health care for ethnic minority groups, and play a ‘gatekeeper’ role within the health care system [26]. An investigation into the issue of ethnic discrimination with respect to general practitioners is therefore relevant [27]. Moreover, most of the research and most of the publications on ethnic discrimination in health care have come from the U.S.A., a societal context that is characterized by a long history of migration and racial segregation. Our study was conducted in Belgium, which provides a different societal, historical, and cultural context, as well as a different organization of the health care system. It has been shown that different contexts may lead to different medical practices in terms of ethnic discrimination. For example, one study concluded that the Danish health care system was equitable for ethnic minority groups [28]. In 2011, the resident foreign population represented 10.2% of the entire population of Belgium [29,30]. The principal ethnic minority groups living in Belgium are Moroccans, Turks, and Congolese [30].

The main aim of this study was to investigate ethnic discrimination in general practice. Our general research hypothesis was that GPs would be more favorable towards non-ethnic minority patients (in-group members) and less favorable towards ethnic minority patients (out-group members). We used the van Ryn model to analyze the effect of patient ethnicity on GPs. This model suggests that there are two major pathways that lead doctors to give different treatment during medical consultations. The first pathway is the cognitive or decisional dimension, the second one is the interpersonal dimension [8]. In this paper, we assess whether patient ethnicity implies systematic differences in the medical care patients receive. In order to test our research hypothesis, we considered the outcome relative to GPs’ decision-making (assessment, recommendation, and prescription) and the outcome relative to the relational aspect of care (time devoted by GPs to examining patients’ medical case history), as suggested by van Ryn. Although the relational dimension of doctoring can be evaluated using many factors, in this study we will measure only one of these: time devoted to patient. It has been argued that consultation duration can be considered to be a generic enabling resource and a reliable indicator of the quality of the relationship and quality of care in general [31-37]. We also looked at whether GPs’ working conditions led to differential medical care for ethnic minority patients.

## Methods

### Study design

Belgian GPs were randomly assigned one of two case vignettes in which every factor except patient ethnicity was identical. The vignette consisted of a written medical description of a hypothetical patient and was provided to GPs within the questionnaire. Patient ethnicity was altered by modifying the name of the patient in the vignette to create two experimental conditions. The non-minority patient condition featured a Belgian patient named Jean-Pierre, while the ethnic minority patient condition featured a Moroccan patient named Mohamed. One group of GPs was therefore given a non-minority patient, while the other was given an ethnic minority patient. The patient in both vignettes presented with the same hypertensive disease and the same environmental information. Patient ethnicity was therefore our main manipulated independent variable.

### Medical vignette

Hypertension is a highly prevalent disease with potentially serious impacts on morbidity and mortality within populations. GPs play a central role in the management of this disease in terms of early detection, treatment, control, and referrals [38,39].

The medical vignette consisted of a high blood pressure disorder (hypertension) with associated risk factors: tobacco use, being overweight and physical inactivity. GPs were also given some medical information: current blood pressure (185/90 mmHg), cardiac frequency (84 bpm), BMI, and results from a blood analysis conducted 15 days previously. The patient’s age was also given, as well as some environmental information about the patient’s family composition (i.e. number of children, marital status), and professional situation (Additional file 1).

### Participants

A sample of 171 general practitioners (GPs) participated in the study (n = 171). The average age of the total sample was 47.03 years old (SD = 13. 76). The gender composition of the sample was 86 males (50. 3%) and 85 females (49. 7%).

Since we used a fictitious patient with fictitious medical data (in the medical vignette), we judged it unnecessary to request approval from any ethics committee. The study instructions stated that participation in the study was free and on a voluntary basis. All of the participants were adults (Family Practitioners). Participants were therefore not required to complete any consent statement.

### Data collection

Data was collected in three different cities (Charleroi, Liège, and Namur) in the Francophone part of Belgium, on three separate occasions between January and March 2012. At each site, all of the GPs were attending an accredited conference day organized by the Belgian Scientific Society of General Medicine. Questionnaires were randomly distributed to GPs as they arrived to register at the conference. The GPs filled in the questionnaires by hand and returned them to the researcher by the end of the day. The GPs were not aware of the study hypotheses, because the survey was presented as research into the “*quality of medical decisions*”. To encourage GPs to participate in the study, a lottery ticket was given to those who completed the questionnaire. The average participation rate for the study was 45%.

### Outcomes measures

The medical activities of GPs can be classified according to two dimensions: relational and medical decisions. The relational dimension of doctoring includes two variables: time devoted to examining medical history and time devoted to examining socio-relational history. Medical decisions include three variables: cardiovascular risk assessment (from 0 to 10, where 0 = no risk and 10 = maximum risk); the medical utility of recommending an electrocardiogram (ECG) (from 0 to 10, where 0 = no utility and 10 = maximum utility), and whether any antihypertensive drug was prescribed (yes/no).

### Statistical analysis

We considered the following characteristics of the GPs: sex, age, ethnicity, type of practice, patient contact per week, hours worked per week, and practice location. Independent samples T-tests were used to assess the effect of the condition (patient ethnicity) on continuous outcomes (time devoted to medical history examination, time devoted to socio-relational history examination, cardiovascular risk assessment, and ECG recommendation). A chi-squared (χ^2^) analysis was used to assess the effect of the condition on the categorical outcome (drug prescription). We also considered that some characteristics of GPs, such as sex, type of practice (solo vs. group), practice location (rural vs. urban), and volume of activity, could moderate the effect of patient ethnicity on the GPs’ medical activities. It has been shown that restrictive working environments diminish the cognitive capacity of GPs and thus increase the likelihood of discrimination [15]. General linear models (GLM) were used to assess interactions between patient ethnicity and GP characteristics for medical activities. All statistical analyses were carried out using IBM SPSS 20.0 for Windows.

## Results

### GP characteristics

Table 1 shows the main characteristics of GPs in our sample, arranged by experimental condition. We found no significant differences in the distribution of GP characteristics between the two experimental conditions. Compared to what is average for the general population of GPs in Belgium, we had more GPs in our sample that were female (49. 7% vs*.* 33%), young (47. 03 vs. 50.25), working in group practices (57. 3% vs*.* 75%) and had slightly lower caseloads.

**Table 1:** Characteristics of Belgian GPs sample by experimental condition (N = 171), 2012

**GPs exposed to**	**Non-minority patient**	**Minority patient**	**N total**	**%**
**Male**	39	47	86	50.3
**Female**	45	40	85	49.7
**Age (yr)**				
26--30	16	15	31	18.2
31--40	14	16	30	17.6
41--50	13	11	24	14.1
51--60	26	28	54	31.8
>61	15	16	31	18.2
**Ethnicity**				
White	79	85	164	95.9
**Type of activity**				
Solo	47	51	98	57.6
Group	36	36	72	42.4
**Patient contacts per week**				
<60	17	17	34	21.5
60--120	49	48	97	61.4
>121	13	14	27	17.1
**Medical activity hours per week**				
8--40	22	19	41	24.9
41--60	52	48	100	60.6
>61	10	14	24	14.5
**Activity localization**				
Rural	32	35	67	40.4
Semi-Urban	29	24	53	31.9
Urban	20	26	46	27.7

### Case vignette comparison by patient ethnicity

Through random distribution, 84 GPs (49. 1% of the total sample) were assigned the non-minority patient vignette and 87 GPs (50. 9% of the total sample) were assigned the ethnic minority patient vignette. We compared the medical activities of GPs according to the experimental conditions (patient ethnicity). Our results revealed no significant difference between the two conditions for the following outcomes: time devoted to examining medical history, cardiovascular risk assessment, and ECG recommendation. However, we found that for ethnic minority patients, GPs devoted less time to examining socio-relational history than for non-minority patients (t = 4.05, p = 0.001). Furthermore, our data suggests that ethnic minority patients are more likely to be prescribed an antihypertensive than non-minority patients (χ^2^ = 5. 51, p = 0.01) (Table 2).

**Table 2:** Differences in medical activities of GPs by experimental exposition to patient’s ethnicity, Belgium, 2012

**Medical activities: Relation & Decisions**	**Ethnic minority patient**			**Non ethnic minority patient**				
	**N**	**Mean/%**	**SD (σ)**	**N**	**Mean/%**	**SD (σ)**	**t/χ²**	**p-value**
**Relation**								
1. Medical history examination (time in minutes)	85	4,05	3,53	84	4,43	2,35	-1,06	0,29
2. Socio-relational history examination (time in minutes)	87	2,11	1,43	84	3,36	2,48	4,05	0,001
**Decisions**								
3. Cardiovascular risk assessment (score from 0 “no risk” to 10 “max risk”)	85	6,32	1,70	82	6,34	1,56	0,05	0,96
4. ECG recommendation (score from 0 “no utility” to 10 “max utility”)	85	5,42	3,04	83	5,13	2,91	-0,06	0,53
5. Drug prescription (Yes/No)	85	70%	–	83	53%	–	5,51	0,01

**Figure 1:**
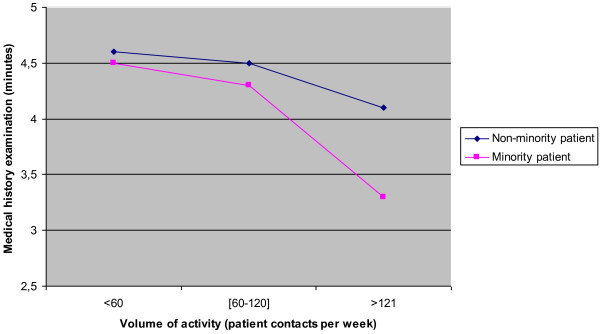
Interaction between patient’s ethnicity and GPs’ volume of activity in estimation of time devoted by GPs to medical history examination, Belgium, 2012.

We found that the number of patient contacts per week interacted significantly with patient ethnicity in terms of time devoted to examining medical history (F = 5.71, p < 0.05). GPs with high volumes of activity were more discriminatory than GPs with lower volumes of activity: the former devoted less time to examining medical history when dealing with an ethnic minority patient than when dealing with a non-minority patient (Figure 1).

We found no other significant interactions between characteristics of GPs and patient ethnicity for medical activities.

## Discussion

This is one of the first experimental studies to investigate ethnic discrimination in general practice. Since GPs are ‘gatekeepers’ to the health care system, this issue is particularly relevant. Based on van Ryn’s model, we hypothesized that patient ethnicity would affect the quality of medical care through (a) biased medical decisions by GPs and (b) biased relational involvement on the part of GPs.

Before beginning a decision-making process, GPs evaluate a patient’s cardiovascular risk*.* We observed that cardiovascular risk assessment was not related to patient ethnicity. The next step is the medical decision and recommendation. Again, ethnicity was not related to ECG procedure recommendation*.* However, we detected that drug prescription was significantly more frequent for ethnic minority patients than for non-minority patients. These results are inconsistent with our first hypothesis, since ethnicity did not lead to an obvious decrease in the quality of medical decisions or recommendations. On the contrary, ethnic minority patients received more pharmacological treatment than non-minority patients. This result is surprising because previous studies have usually described fewer drug prescriptions for ethnic minority patients suffering from cardiovascular diseases than for non-minority patients [40]. So why did GPs in our study prescribe more drugs for ethnic minority patients, when the cardiovascular risk was equivalent in both groups? One possible explanation is that GPs may consider ethnic minority patients to belong to an at-risk population, due to their ethnicity, and therefore prescribe medication as a protective measure. GPs may have considered that most cardiovascular risk factors are more prevalent in ethnic minority populations than in the rest of the population [41]. For example, in Belgium, ethnic minorities are more likely to be living in a poorer socio-economic environment than the general population [42,43]. The general health status of ethnic minorities in Belgium is worse than that of the general population [44]. Ethnic minorities are more exposed to cardiovascular risk factors and therefore incur higher cardiovascular morbidity and mortality [45,46]. Research indicates that doctors over-apply population statistics to individual patients [47]. Population statistics may have a similar function to stereotypes in decision-making by doctors. Our results may therefore suggest that decisions made by GPs for ethnic minority patients are based on over-generalizations rather than on the actual individual assessment of the patient. This phenomenon suggests that GPs are more likely to perceive ethnic minority patients (out-group members) in terms of their group stereotype and pay less attention to their individual characteristics. An alternative explanation could be that GPs may prescribe drugs in order to avoid becoming involved with lifestyle modification, which is a more demanding clinical activity requiring more inter-personal communication. For example, studies show that doctors with a longer mean consultation time explore the psychosocial dimension of illness in greater depth and prescribe fewer medications than doctors with shorter consultation times. The same authors also showed that, interestingly, the doctors who were devoting more time to patients were those who were experiencing higher stress levels associated with “running behind schedule” [33,34]. We speculate that, when facing ethnic minority patients, GPs may devote less time to them and prescribe them more medication as a coping strategy to avoid engaging in doctor-patient relations.

As regards the relational dimension of doctoring, ethnicity had no effect on the amount of time devoted by GPs to examining the patient’s medical history. However, we found that GPs experiencing high volumes of activity devoted significantly less time to medical history examination for ethnic minority patients than did GPs with lower volumes of activity. This result is consistent with previous research that indicated that time pressure, cognitive load, necessity of prompt decisions, task complexity, busyness, distraction, fatigue, and anxiety increase the likelihood of stereotype usage [48-52]. Our findings therefore support the hypothesis that ethnic discrimination is likely to be context-dependent. Baron and Pfeffer showed that organizational practices mediate the cognitive biases and stereotypes of actors [53]. In addition, Reskin states that “*the proximal cause of most discrimination is in the personal practices of work organization that polarize the biasing effects of automatic cognitive processes*” ( [54], p.320). The underlying idea is that discrimination is expressed only if certain organizational conditions are met [55]. We observed that the amount of time devoted by GPs to examining socio-relational history was significantly lower for ethnic minority patients. These results are consistent with Roter and Johnson’s research, which revealed that doctors engaged in less psychosocial talk and more biomedical information-giving during inter-ethnic consultations [22,23]. They are also consistent with Cooper, who found that race-discordant medical interactions were significantly shorter compared to race-concordant medical interactions [56]. When interacting with ethnic minority patients, GPs are thus focused on medical aspects of doctoring and put aside the socio-relational dimensions of doctoring. This is supported by previous findings. Doctors engaged in inter-ethnic consultations have been shown to be more reluctant to engage in partnership-building and emotional considerations [25]. The literature on doctor-patient communication supports the idea that white doctors are less comfortable when interacting with patients of other ethnic groups [9,25].

There were at least three limitations to our study. Firstly, the participation rate was low (45%). The representativeness of our sample is therefore limited. Our GP sample was over-representative of younger, female, and group-practice GPs when compared with the overall population of GPs in Belgium. This may mean that we surveyed a more “migrant friendly” group of GPs, since younger and female GPs have been shown to be more patient-centered and more concerned about vulnerable patients, compared to older, male doctors [57]. Secondly, case vignettes are not real clinical situations and it is difficult to determine whether the relational engagement with patient and medical decisions by GPs in case vignettes represent their true clinical activity. Thirdly, the relational dimension of doctoring involves more than the time devoted to the patient, which is only one of many aspects. Further studies, using more ethnographic methods, could consider other factors such as participatory style, warmth, empathy, and information giving/seeking, which are also essential aspects of the relational dimension of doctoring.

## Conclusion

Good doctor-patient relations are essential for the effective management of chronic illness in general practice. In this study, we found that GPs self-report devoting less time to ethnic minority patients than to non-ethnic minority patients. This may suggest that ethnic discrimination in general practice occurs primarily through the relational dimension of doctoring. However, as our study design was experimental, our results require confirmation through the use of observational data and routine health care databases. We also observed that GPs in a constraining environment (high rate of patient contact) devoted less time to examining medical history for ethnic minority patients. This last finding suggests that ethnic discrimination is associated with resource allocation priorities and is therefore a context-dependent phenomenon. Further research should, firstly, aim to corroborate these ethnic differences in medical decisions with routine databases that allow GPs’ prescriptions, patient clinical status, and patient ethnicity to be tracked. Secondly, further research should seek to understand why GPs engage less in the relational dimension of doctoring with ethnic minority patients. Third, could also attempt to identify key contextual variables that enhance the risk of ethnic discrimination. Further research is also necessary to replicate and confirm our findings. Research into the issue of ethnic discrimination in general practice potentially has important public health benefits. GPs are the gatekeepers to specialty care and play an important role in patient pathways. Discrimination may also magnify ethnic inequalities in health. Our study highlights the risk of inappropriate drug use in some ethnic-specific encounters.

## Abbreviations

GP: General practitioner; GPs: General practitioners; ECG: Electrocardiogram.

## Competing interests

All authors declare no competing interests.

## Authors’ contribution

BL conceived the study, performed the statistical analysis, and drafted the manuscript. VL participated in the design of the study and helped to draft the manuscript. CR and PvM assisted with the interpretation of the data. All authors participated in the analysis and read and approved the final manuscript.

## Pre-publication history

The pre-publication history for this paper can be accessed here:

http://www.biomedcentral.com/1471-2296/15/89/prepub

## Supplementary Material

Additional file 1 Medical vignette used in the study. Click here for file
