# Supplementary material for: General practice and ethnicity: an experimental study of doctoring
Source: BMC Fam Pract. 2014 May 9;15:89. doi: 10.1186/1471-2296-15-89 (PMC4101847; doi:10.1186/1471-2296-15-89)
Supplement: Additional file 1 — Medical vignette used in the study. [file 1471-2296-15-89-S1.docx]

**Medical vignette used in the study to create two experimental conditions**

- 1. **Non-minority patient condition**

**Medical vignette**

Jean-Pierre B. is 42 years old. He works in an electric company. Jean-Pierre B. is married and has two children: Anaïs (10 years old) and Bruno (8 years old).

Jean-Pierre B. is referred to you by the occupational doctor who measured a blood pressure (BP) of 170/95 mmHg last week. Today, you identify a BP of 185/90 mmHg and a heart rate 84 beats/min. The BP is controlled at the end of the consultation and you observe a value of 183/92 mmHg.

During the consultation, you learn that Jean-Pierre B. does not consume alcohol. He smokes about 15 cigarettes a day and undertakes no physical activity.

Jean-Pierre B. is not currently undergoing any medical treatment.

**Medical data:**

- Jean-Pierre B. weighs 92 kg and is 1.82 metres tall

- His waist circumference is 104 cm

- His BMI is 28

- A blood test of 15 days ago reveals the following parameters:

[Jean-Pierre B., born in Brussels on 17 November, 1969]

- 1. **Minority patient condition**

**Medical vignette**

Mohamed B. is 42 years old. He works in an electric company. Mohamed B. is married and has two children: Saïda (10 years old) and Bachir (8 years old).

Mohamed B. is referred to you by the occupational doctor who measured a blood pressure (BP) of 170/95 mmHg last week. Today, you identify a BP of 185/90 mmHg and a heart rate 84 beats/min. The BP is controlled at the end of the consultation and you observe a value of 183/92 mmHg.

During the consultation, you learn that Mohamed B. does not consume alcohol. He smokes about 15 cigarettes a day and undertakes no physical activity.

Mohamed B. is not currently undergoing any medical treatment.

**Medical data:**

- Mohamed B. weighs 92 kg and is 1.82 metres tall

- His waist circumference is 104 cm

- His BMI is 28

- A blood test of 15 days ago reveals the following parameters:

[Mohamed B., born in Casablanca on 17 November, 1969]
